# The rho kinase inhibitor Y-27632 improves motor performance in male SOD1^G93A^ mice

**DOI:** 10.3389/fnins.2014.00304

**Published:** 2014-10-07

**Authors:** René Günther, Kim-Ann Saal, Martin Suhr, David Scheer, Jan Christoph Koch, Mathias Bähr, Paul Lingor, Lars Tönges

**Affiliations:** ^1^Department of Neurology, University Medicine GöttingenGöttingen, Germany; ^2^Nanoscale Microscopy and Molecular Physiology of the Brain, Cluster of Excellence 171–DFG Research Center 103 (CNMPB)Göttingen, Germany

**Keywords:** ROCK, Y-27632, SOD1^G93A^, ALS, neuroprotection

## Abstract

Disease progression in amyotrophic lateral sclerosis (ALS) is characterized by degeneration of motoneurons and their axons which results in a progressive muscle weakness and ultimately death from respiratory failure. The only approved drug, riluzole, lacks clinical efficacy so that more potent treatment options are needed. We have identified rho kinase (ROCK) as a target, which can be manipulated to beneficially influence disease progression in models of ALS. Here, we examined the therapeutic potential of the ROCK inhibitor Y-27632 in both an *in vitro* and in an *in vivo* paradigm of motoneuron disease. Application of Y-27632 to primary motoneurons *in vitro* increased survival and promoted neurite outgrowth. *In vivo*, SOD1^G93A^ mice were orally treated with 2 or 30 mg/kg body weight of Y-27632. The 2 mg/kg group did not benefit from Y-27632 treatment, whereas treatment with 30 mg/kg resulted in improved motor function in male mice. Female mice showed only limited improvement and overall survival was not modified in both 2 and 30 mg/kg Y-27632 groups. In conclusion, we provide evidence that inhibition of ROCK by Y-27632 is neuroprotective *in vitro* but has limited beneficial effects *in vivo* being restricted to male mice. Therefore, the evaluation of ROCK inhibitors in preclinical models of ALS should always take gender differences into account.

## Introduction

Amyotrophic lateral sclerosis (ALS) is a neuromuscular disease which is characterized by degeneration of the first and second motoneuron resulting in severe progressive muscle weakness and most often death from respiratory insufficiency within only a few years after initial diagnosis. It is known to be a neurodegenerative process that comprises the loss of motoneuronal cells, of their axons and neuromuscular junctions but also non-neuronal cell populations contribute to the progression of disease. Because first pathological signs of motoneuron degeneration are observed at the distal end of its axonal projections some authors consider ALS to be a distal axonopathy (Fischer and Glass, 2007). The molecular and pathophysiological mechanisms of the disorder are multifactorial and include protein aggregation, oxidative stress, excitotoxicity, mitochondrial dysfunction, and impaired axonal transport (Ferraiuolo et al., 2011). Moreover, there is a strong involvement of glial cells such as astrocytes, microglia and oligodendrocytes, which may determine the onset and the dynamics of disease progression (Philips and Robberecht, 2011).

Apart from the majority of sporadic forms, there exist at least 10% of familial forms of ALS (fALS). Among these, genetic mutations in the super oxide dismutase gene 1 (SOD1) were identified first and today represent about 20% of all familial forms (Rosen et al., 1993). Recently, many other familial cases have been identified which exhibit mutations e.g., in Fused in sarcoma (FUS), TAR DNA-binding protein 43 (TDP-43) or exhibit an intronic hexanucleotide repeat in the gene C9orf72. The C9orf72 repeat expansion is considered to be the most frequent genetic alteration in fALS patients but has also been identified in many patients with sporadic ALS (sALS). Therefore, longstanding concepts of the cause and molecular pathology in ALS are currently re-evaluated (Turner et al., 2013).

The fALS gene mutations have permitted the generation of transgenic mouse models of ALS. Because it closely recapitulates human disease progression the SOD1^G93A^ mouse model has evolved as a standard model for the evaluation of therapeutic effects in preclinical studies (Turner and Talbot, 2008). A variety of neuroprotective agents have been examined to both delay the onset of clinical disease and to prolong the disease course ultimately extending life span in animal models, but only the glutamate antagonist riluzole has reached clinical use so far (Benatar, 2007). However, this agent has very limited benefits in ALS patients (Stewart et al., 2001).

Rho kinases (ROCK) are serine/threonine kinases that regulate cytoskeleton structure through phosphorylation of LIM kinases, myosin light chain or the ezrin/radixin/moesin protein complex (Tonges et al., 2011). Our group and others have shown that inhibition of ROCK with pharmacological small molecule inhibitors is not only able to improve the regenerative response in the lesioned CNS (Lingor et al., 2008; Planchamp et al., 2008; Bermel et al., 2009), but also exerts neuroprotection via activation of intracellular cell survival signaling such as the Akt pathway (Tonges et al., 2012; Takata et al., 2013). In addition, we have recently demonstrated that the use of fasudil, a small-molecule ROCK inhibitor of the isoquinoline series, strongly modulates microglial function in SOD1^G93A^ female mice which may be a major component of the therapeutic effect (Tonges et al., 2014).

Based on these results, we have designed both an *in vitro* and an *in vivo* study to evaluate the neuroprotective potential of ROCK inhibition with a more specific ROCK inhibitor, the 4-aminopyridine derivative Y-27632.

## Materials and methods

### Motoneuron preparation

Lumbar spinal cord mouse motoneurons (MN) of E11.5–E13.5 embryos of wild type (WT) mice (B6/SJL background) were generated applying a preparation technique adapted from Wiese et al. (2010). In the case of later immunoblot and cell toxicity analysis the panning step was omitted in order to obtain a higher cell yield. In all cases, cells were collected and seeded on poly-L-ornithine/laminin coated cover slips in MN complete medium containing Neurobasal medium without (2)L-Glutamine (Invitrogen, Darmstadt, Germany), supplemented with 2% horse serum (Linaris, Wertheim, Germany), B27-supplement (Invitrogen, Darmstadt, Germany), BDNF (final concentration 10 ng/mL; Tebu Bio, Offenbach, Germany), and CNTF (final concentration 10 ng/mL; Tebu Bio, Offenbach, Germany) at a density of 25,000 cells/cm^2^.

### Motoneuron culture, quantification of cellular survival, cytotoxicity and neurite length

MN were cultured in MN complete medium and supplemented with Y-27632 (final concentration 10 μM; Sigma-Aldrich, St. Louis, Mo) or the respective amount of vehicle every second day. Additionally, BDNF and CNTF (final concentration 10 ng/mL, both Tebu Bio) were supplemented every second day. Motoneuron survival was assessed by counting ChAT-immunopositive cells after fixation and immunocytochemistry on DIV4.

Cytotoxicity assays were also done on DIV4. Here, a bioluminescence-based assay for the release of adenylate kinase (AK) from lesioned cells was applied according to the manufacturer's instructions (ToxiLight^®^, Lonza, Wakersville, USA). Briefly, the amount of adenylate kinase (AK) was determined in the culture medium by measuring the AK-dependent conversion of ADP to ATP and subsequent light emission by luciferase with a luminometer (Wallac 1450 MicroBeta Trilux, PerkinElmer, Shelton, USA).

The length of all neurites of ChAT-immunopositive cells was evaluated semi-automatically using the axon tracing module of “Image J” (Free Java software provided by the National Institutes of Health, Bethesda, Maryland, USA) and was divided by the numbers of ChAT-immunopositive cells in order to obtain neurite length/cell. Results were expressed in relation to vehicle treated cells. The immunofluorescence-based quantification of intracellular ROCK2 protein was done by measuring mean fluorescence intensity values in MN perikarya with “ImageJ.” For MN survival at least three and for neurite outgrowth at least two independent experiments were evaluated. The quantification of perikaryal ROCK2 was done in at least 6 MN per treatment condition.

### Immunocytochemistry

For MN immunolabeling, cells were fixed in PFA 4% for 10 min at room temperature (RT, 22°C), permeabilized with 100% ice-cold acetone (AppliChem, Darmstadt, Germany) 10 min at −20°C, washed twice with PBS and blocked with 10% normalized goat serum 10 min at RT. Probes were incubated with the primary antibodies (rabbit anti ChAT 1:50, Millipore, Schwalbach, Germany; goat anti-ROCK2 1:50, Santa Cruz Biotechnology Inc., Heidelberg, Germany) for 1 h at 37°C or were fixed in PFA 4% for 10 min at room temperature (RT, 22°C), washed twice with PBS, incubated 30 min in 25 mM Glycine in PBS (Applichem, Darmstadt, Germany), permeabilized and blocked with 10% horse serum, 5% BSA, 0,3% Triton, 25 mM Glycine in PBS at RT for 1 h and then incubated with primary antibodies (rabbit anti ChAT 1:50, Millipore, Schwalbach, Germany; mouse anti-MAP2 1:500 Chemicon/Millipore, Schwalbach, Germany) over night at 4°C.

Following three PBS washes, Cy3- or Cy2-labeled secondary antibodies (1:250, Dianova, Hamburg, Germany) were applied for 1 h at room temperature. After another three PBS washes, cells were then nuclear counter-stained with DAPI (4,6-diamidino-2-phenylindole) (Sigma, Taufkirchen, Germany), optionally incubated with additional Rhodamine-Phalloidin 1:500 in PBS (Invitrogen, Eugene, Oregon, USA) and mounted in Mowiol (Hoechst, Frankfurt, Germany). Immunolabeled fluorescent cells were imaged on a Zeiss Axioplan 2 fluorescence microscope equipped with a CCD camera and AxioVision software (Zeiss, Göttingen, Germany). For evaluation of survival and neurite outgrowth of ChAT-immunopositive cells, micrographs were taken with a 10× objective of at least four random visual fields per culture well and of at least two wells per condition.

### Motoneuron cell culture lysis and immunoblotting

Motoneuron cultures were plated at a density of 25,000 cells/cm^2^, cultured in MN complete medium and supplemented with Y-27632 (final concentration 10 μM; Sigma-Aldrich, St. Louis, Mo) or the respective amount of vehicle every second day. After 5 days cells were lysed with a cell lysis buffer (Thermo Scientific™ Pierce™ RIPA Buffer, Thermo Fisher Scientific Inc., Waltham, MA USA) plus protease inhibitors (“Complete tablets,” Roche, Basel, Switzerland) and phosphatase inhibitor (“PhosSTOP,” Roche, Basel, Switzerland). The protein content of all cell samples was determined using Bradford (Biorad, Munich, Germany) and equal amounts of protein (20 μg) were separated on a sodium dodecyl sulfate–polyacrylamide gel electrophoresis.

Proteins were then electrotransferred onto a PVDF membrane (Applichem, Darmstadt, Germany) and blocked with 5% milk in Tris-buffered saline/Tween-20 (TBS-T) for 1 h. Membranes were then incubated with the primary antibodies goat anti-ROCK2 1:50 (Santa Cruz Biotechnology Inc., Heidelberg, Germany) and mouse anti-GAPDH 1:1000 (HyTest Ltd, Turku, Finland) in 5% milk TBS-T or 5% BSA TBS-T over night at 4°C. This was followed by incubation with corresponding horseradish peroxidase-coupled secondary antibodies (1:1000 for 1 h at room temperature; Dianova, Hamburg, Germany). ECL-Plus reagent (Amersham, Arlington Heights, IL, USA) was applied on the membrane and the chemiluminescence was visualized on an Amersham Hyperfilm ECL (GE Healthcare, Chalfont St Giles, GB).

### Animal housing, breeding, genotyping and application of Y-27632

All animal experiments were carried out according to the regulations of the local animal research council and legislation of the State of Lower Saxony. High-copy number B6/SJL-Tg(SOD1^*^G93A)1Gur/J (labeled as SOD1^G93A^ in the following text) (Gurney et al., 1994) were obtained from Jackson Labs (Stock Number 002726; Bar Harbor, USA). The colony was maintained by crossing B6/SJL males harboring the transgene with wild-type B6/SJL females. Housing of animals was performed under a 12 h light/12 h dark cycle with free access to food and water.

For genotyping, tail biopsies of 14-day-old pups were subjected to a standardized PCR protocol (Jackson Labs). Probe sequences were: hSOD1-forward, CATCAGCCCTAATCCATCTGA; hSOD1-reverse, CGCGACTAACAATCAAAGTGA; Interleukin 2-forward, CTAGGCCACAGAATTGAAAGATCT, Interleukin 2-reverse, GTAGGTGGAAATTCTAGCATCATCC).

Y-27632 was administered at a concentration of 2 mg/kg body weight per day (subsequently termed as Y2) or at a concentration of 30 mg/kg body weight per day (Y30) in drinking water according to previous studies with Y-27632 in animal disease models (Nagaoka et al., 2005). Control groups received drinking water without supplementation (Veh).

### Clinical and behavioral animal testing

#### Experimental groups

At day of life 50 (d50) mice were allocated to the different treatment groups. In a first trial a Y-27632 dose of 2 mg/kg (Y2) was compared with vehicle (Veh). In a second trial a Y-27632 dose of 30 mg/kg (Y30) was compared with vehicle (Veh). Both trials were performed in an observer-blinded fashion and groups were constituted to minimize inter-group variability by matching of animals with respect to body weight, age and litter.

#### Assessment of neurological score, body weight and survival

Neurological scores and body weight were assessed every 3 days for each mouse from 50 days of age. The neurological score employed a scale of 0 (worst) to 4 (best) (Weydt et al., 2003). Mice showing an inability to right themselves 30 s after being placed on a side or having a 25% loss of their initial body weight were scored as “dead,” and were euthanized using carbon dioxide.

#### Rotarod test

After training for three consecutive times of at least 180 s at a constant speed of 15 r.p.m. the time for which an animal could remain on the rotating rod (Ugo-Basile, Comerio, Italy) was measured (Gunther et al., 2012). The mean of three trials was recorded for each animal.

#### Hanging wire test

Animals were placed on a custom-made wire lid (0.8 cm spacing) and cautiously turned upside down, 60 cm above a straw-covered bottom (Gunther et al., 2012). After training for three consecutive times of at least 180 s the latency to fall was measured. The mean of three trials was recorded.

### Statistical analysis

For *in vitro* experiments, the comparison between two groups was carried out using an unpaired Student's *t*-test. Behavioral data were subjected to a multifactorial ANOVA analysis and post hoc Fisher's test. Survival data were analyzed using Kaplan-Meier survival fit analysis with Log-Rank tests for statistical significance. Statistical analyses were performed using Statistica 10 (StatSoft, Hamburg, Germany) and Kyplot 2.0 (KyensLab Inc, Tokyo, Japan). Data are represented as mean ± standard error of the mean. Significances are ^*^P < 0.05; ^**^P < 0.01; ^***^P < 0.001.

## Results

### Pharmacological inhibition of ROCK with Y-27632 increases survival and promotes neurite outgrowth of motoneurons *in vitro*

**Figure 1:**
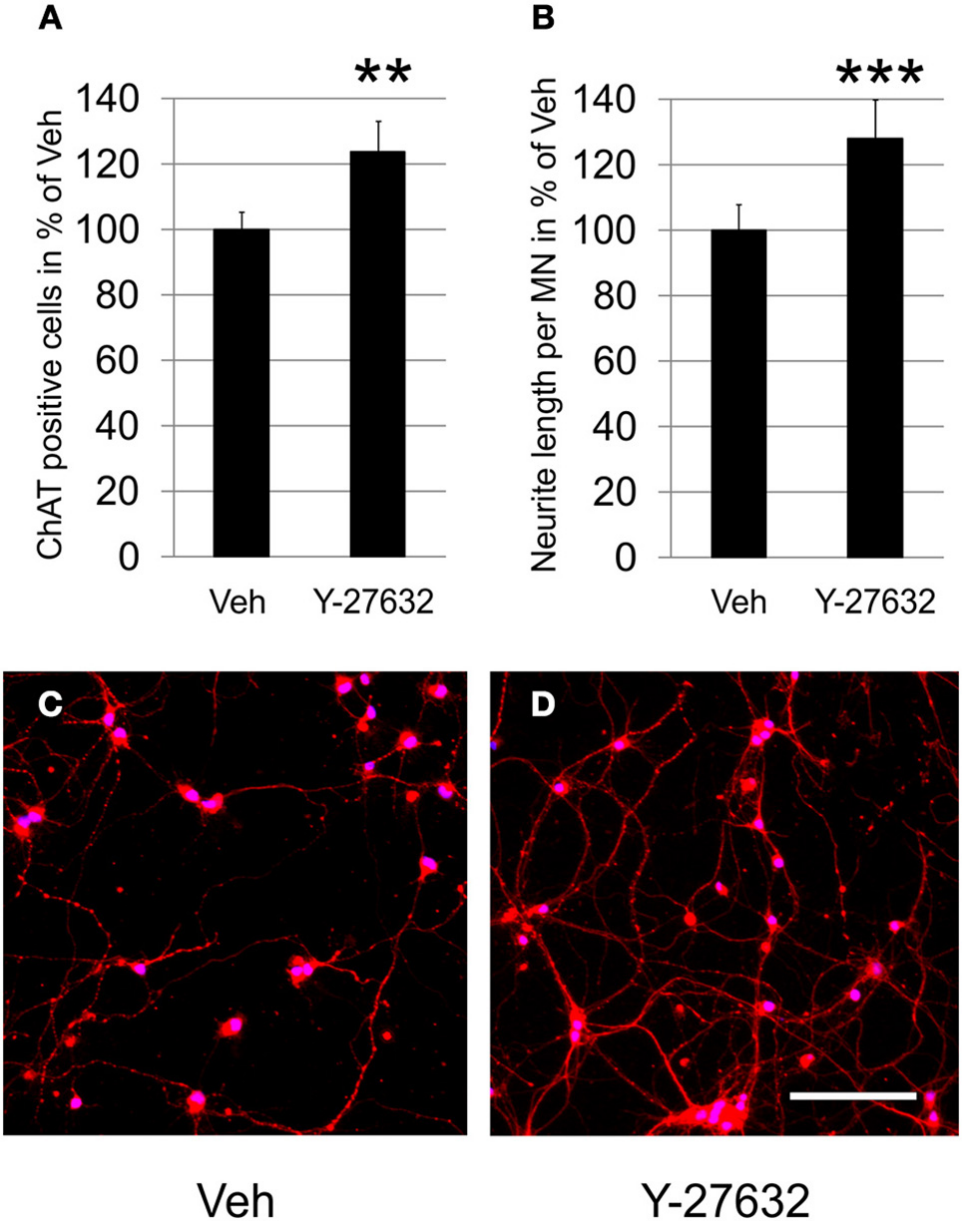
**Y-27632 increases survival of dissociated MN and promotes neurite outgrowth. (A)** Relative numbers of ChAT-immunopositive MN per visual field on DIV4 in cultures treated with Veh or with Y-27632 (10 μM) (*n* = 3 MN cultures, bars represent means ± s.e.m.; ^**^P < 0.005, according to unpaired Student's *t*-test). **(B)** Relative neurite length per ChAT-immunopositive MN treated as in **(A)** (*n* = 2 MN cultures, bars represent means ± s.e.m.; ^***^P < 0.001, according to unpaired Student's *t*-test). **(C,D)** Representative photomicrographs of MN immunolabeled with ChAT (Cy3, red) and treated with Veh **(C)** or Y-27632 (10 μM) **(D)** (nuclear counter-stain with DAPI, blue). Scale bar, 200 μm.

Based on findings that ROCK inhibition is able to attenuate cell death in CNS neurons we examined the neuroprotective potential of Y-27632 in primary MN in dissociated cell culture. In comparison to the number of surviving MN after 4 days *in vitro* in the vehicle treated culture which was set to 100% (100.0 ± 5.3%), the Y-27632 treated culture showed significantly higher numbers of MN (123.7 ± 9.5%) (Figure 1A). This protective effect was also observed in a cytotoxicity assay in which the release of adenylate kinase was reduced after Y-27632 treatment (Supplementary Figure 1).

In the same cultures we measured the total neurite net and calculated neurite length per MN in order to remain independent from MN cell numbers. Compared to the vehicle control treatment, which was set to 100% (100.0 ± 7.7%), application of 10 μM Y-27632 resulted in a significantly increased neurite length of ChAT-immunopositive motoneurons (128.0 ± 11.8%) (Figures 1B–D). In a dose-escalating experiment we observed that a 10 μM final dose of Y-27632 was superior to 5-fold lower or higher doses, which were not significantly promoting neurite outgrowth (Supplementary Figure 2).

**Figure 2:**
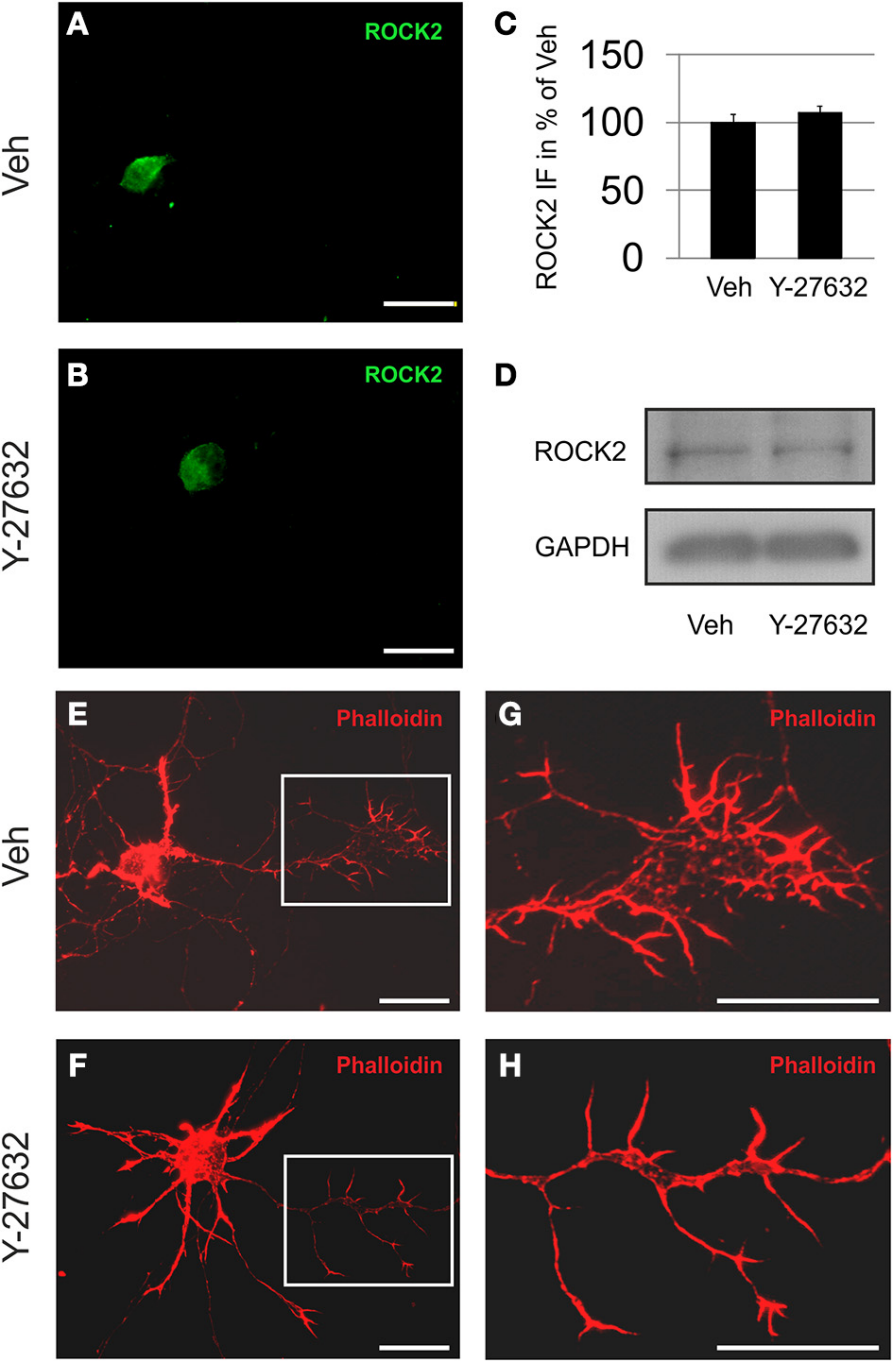
**Motoneurons express ROCK2 protein**. Depicted are MN treated with vehicle **(A,E,G)** or Y-27632 (10 μM) **(B,F,H)**. **(A,B)** ROCK2 is localized primarily in the cytoplasm in both vehicle and Y-27632-treated MN. **(C)** Quantification of perikaryal ROCK2 immunofluorescence (IF) in MN (*n* = 6 cells, bars represent means ± s.e.m., according to unpaired Student's *t*-test). **(D)** Immunoblots of motoneuron cell cultures for ROCK2 and GAPDH. **(E,F)** MN harbor high amounts of f-actin (rhodamine-phalloidin, red). **(G,H)** Magnifications of growth cone areas as depicted in **(E,F)**. Scale bar, 25 μm.

The presence of ROCK2 in motoneurons was demonstrated both immunocytochemically in ChAT immunopositive cells and by immunoblot of cell culture lysates. Here, treatment with Y-27632 was not found to influence the amount of ROCK2 protein being expressed. However, the application of Y-27632 resulted in altered growth cone morphology with prolonged processes if compared to vehicle treated cells (Figure 2).

### Weight dynamics and survival of Y-27632 treated mice

In a proof-of-principle approach, we used the SOD1^G93A^ mouse model to assess the effects of oral Y-27632 treatment with 2 mg/kg body weight (Y2) and 30 mg/kg body weight (Y30) compared against vehicle (Veh) in a presymptomatic trial design starting at day of life 50 (d50).

**Figure 3:**
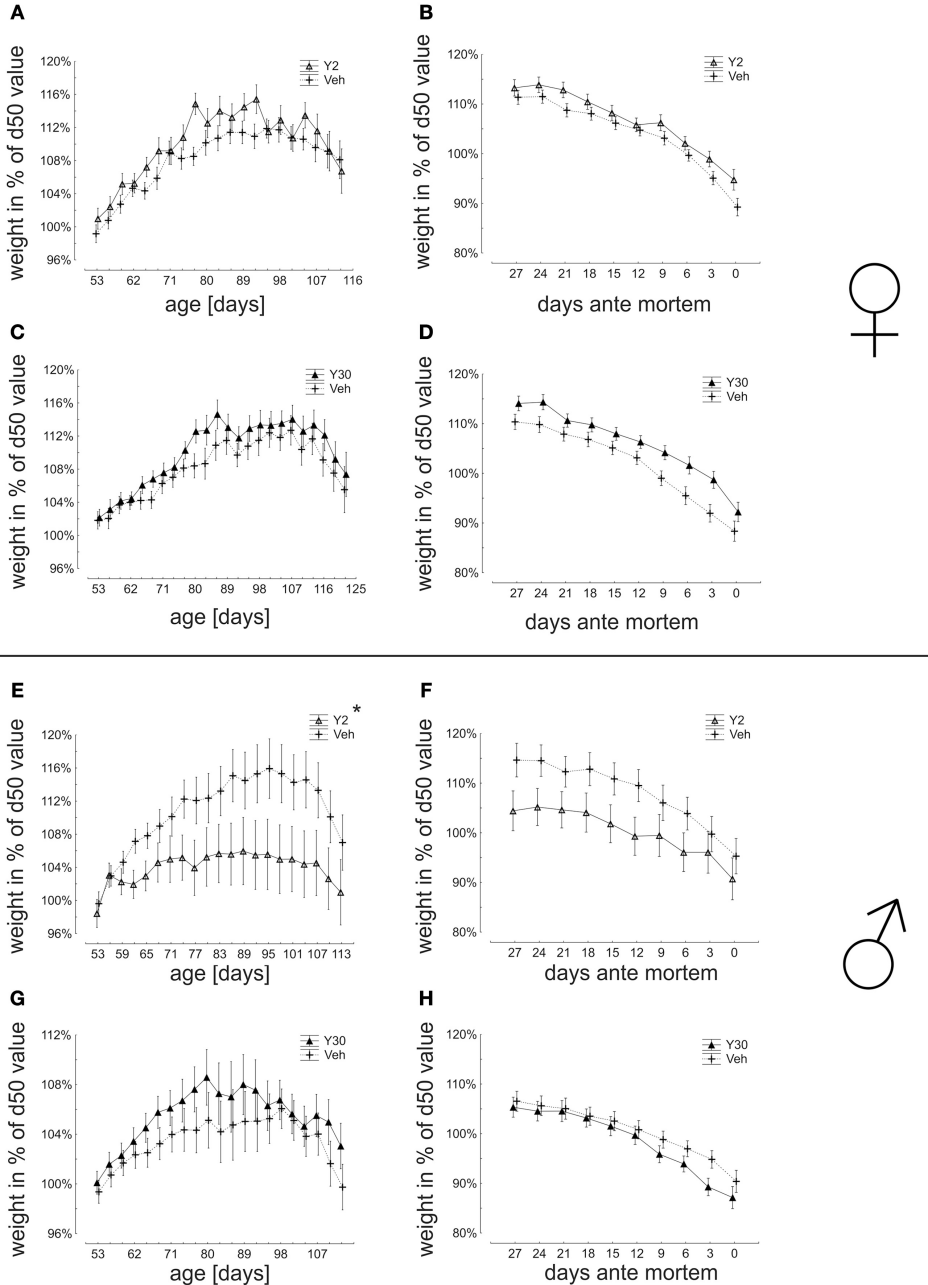
**Weight dynamics in relation to body weight at day 50 (start of presymptomatic treatment) in SOD1^G93A^ mice treated with 2 or 30 mg/kg body weight Y-27632. (A–D)** Weight dynamics in female mice treated with Y-27632 from d50 in a dosage of 2 mg/kg **(A,B)** (Y2 *n* = 13; Veh *n* = 18) or 30 mg/kg **(C,D)** (Y30 *n* = 13; Veh *n* = 12). **(E–H)** Weight dynamics in male mice treated with Y-27632 from d50 in a dosage of 2 mg/kg **(E,F)** (Y2 *n* = 10; Veh *n* = 14) or 30 mg/kg **(G,H)** (Y30 *n* = 8; Veh *n* = 8). Statistics was done applying multifactorial ANOVA and *post-hoc* Fisher's test. ^*^P < 0.05.

Female mice, which have been treated with Y30, had a slightly higher weight in an advanced age, which was, however, not reaching significance. The Y2 female treatment group was not different from vehicle control (Figures 3A–D). Male animals treated with Y2 did not gain as much maximal weight as controls. The Y30 male treatment group was not different from the control (Figures 3E–H).

**Figure 4:**
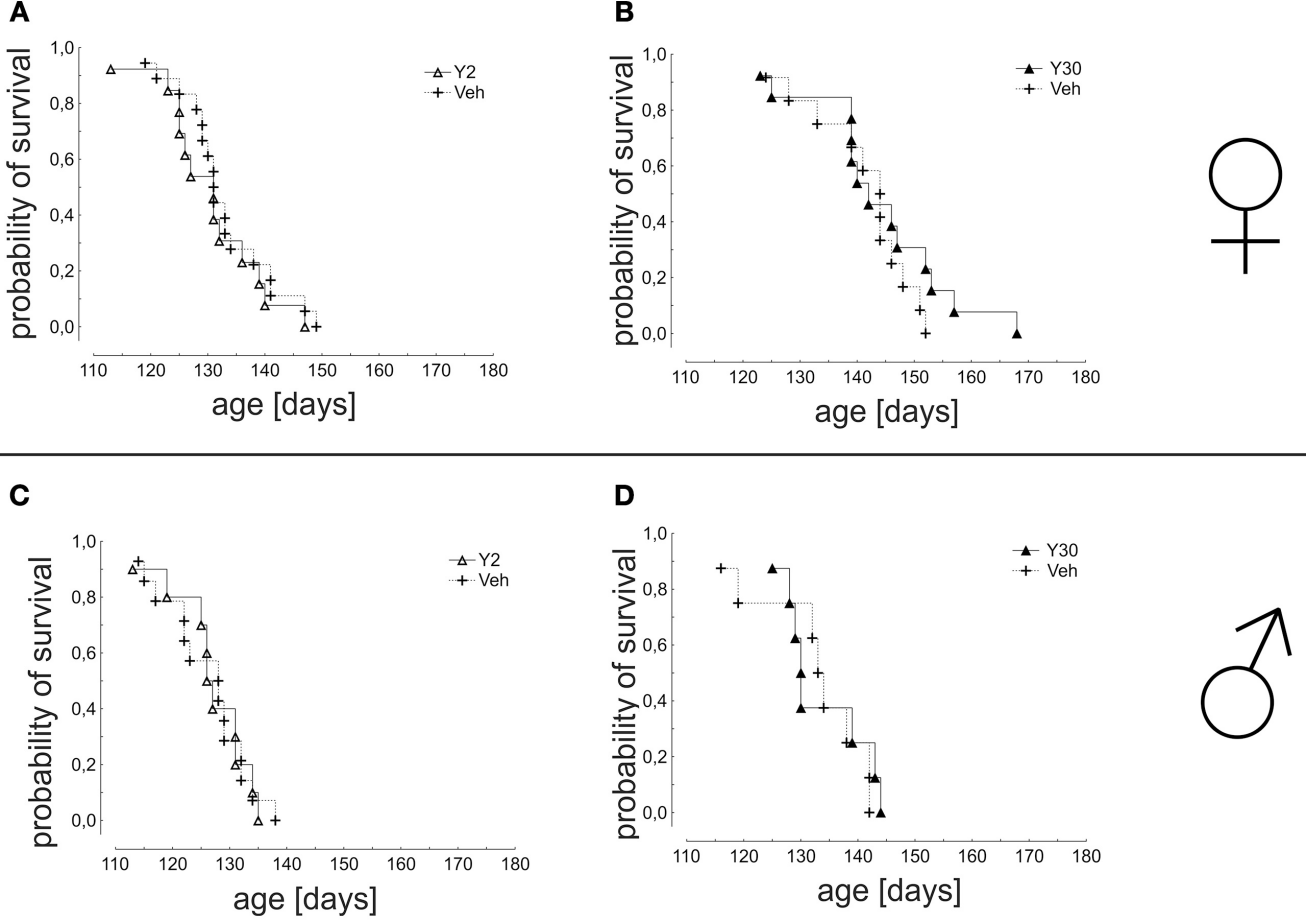
**Survival in SOD1^G93A^ mice treated with 2 or 30 mg/kg body weight Y-27632**. Depicted is a Kaplan-Meier curve for cumulative probability of survival for female mice treated with 2 mg/kg **(A)** (Y2 *n* = 13; Veh *n* = 18) or 30 mg/kg Y-27632 **(B)** (Y30 *n* = 13; Veh *n* = 12) and for male mice treated with 2 mg/kg **(C)** (Y2 *n* = 10; Veh *n* = 14) or 30 mg/kg Y-27632 **(D)** (Y30 *n* = 8; Veh *n* = 8). Days ante mortem refers to the time point before death of an individual animal measured in days. Statistics was done applying Kaplan-Meier survival fit analysis with Log-Rank test.

The analysis of mean survival times did not show a significant improvement by Y-27632 treatment for any group (females: Veh 132,8 ± 1,9 days vs. Y2 130,8 ± 2,4 days; Veh 141,2 ± 2,5 days vs. Y30 143,9 ± 3,4 days; males: Veh 125,9 ± 1,9 days vs. Y2 125,5 ± 2,2 days; Veh 132 ± 3,5 days vs. Y30 133,5 ± 2,6 days) (Figure 4).

### Neurological scoring and motor behavior are improved in male mice treated with high dose of Y-27632

**Figure 5:**
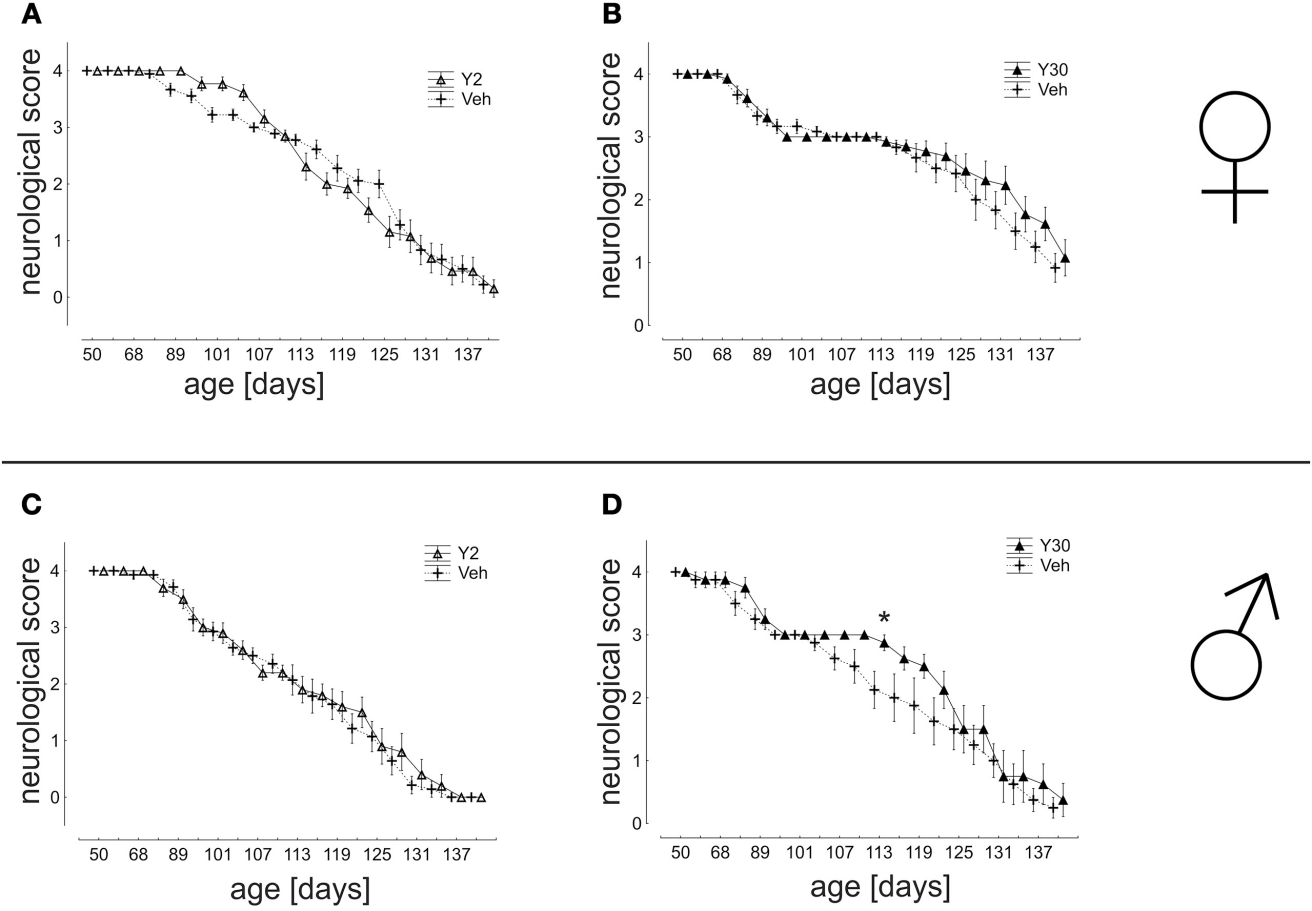
**Neurological score in SOD1^G93A^ mice treated with 2 or 30 mg/kg body weight Y-27632. (A,B)** Depicted are scores for female mice treated with Y-27632 from d50 in a final dosage of 2 mg/kg **(A)** (Y2 *n* = 13; Veh *n* = 18) or 30 mg/kg **(B)** (Y30 *n* = 13; Veh *n* = 12). **(C,D)** Scores for male mice treated with Y-27632 from d50 in a final dosage of 2 mg/kg **(C)** (Y2 *n* = 10; Veh *n* = 14) or 30 mg/kg **(D)** (Y30 *n* = 8; Veh *n* = 8). Statistics was done applying multifactorial ANOVA and *post-hoc* Fisher's test. ^*^P < 0.05.

In a detailed analysis of disease progression based on the neurological score we found nearly no significant differences between Y-27632 and Veh-treated groups. A significant effect was, however, seen in Y30 treated male mice which showed improved values for the neurological score from d107 until d122 (Figure 5).

**Figure 6:**
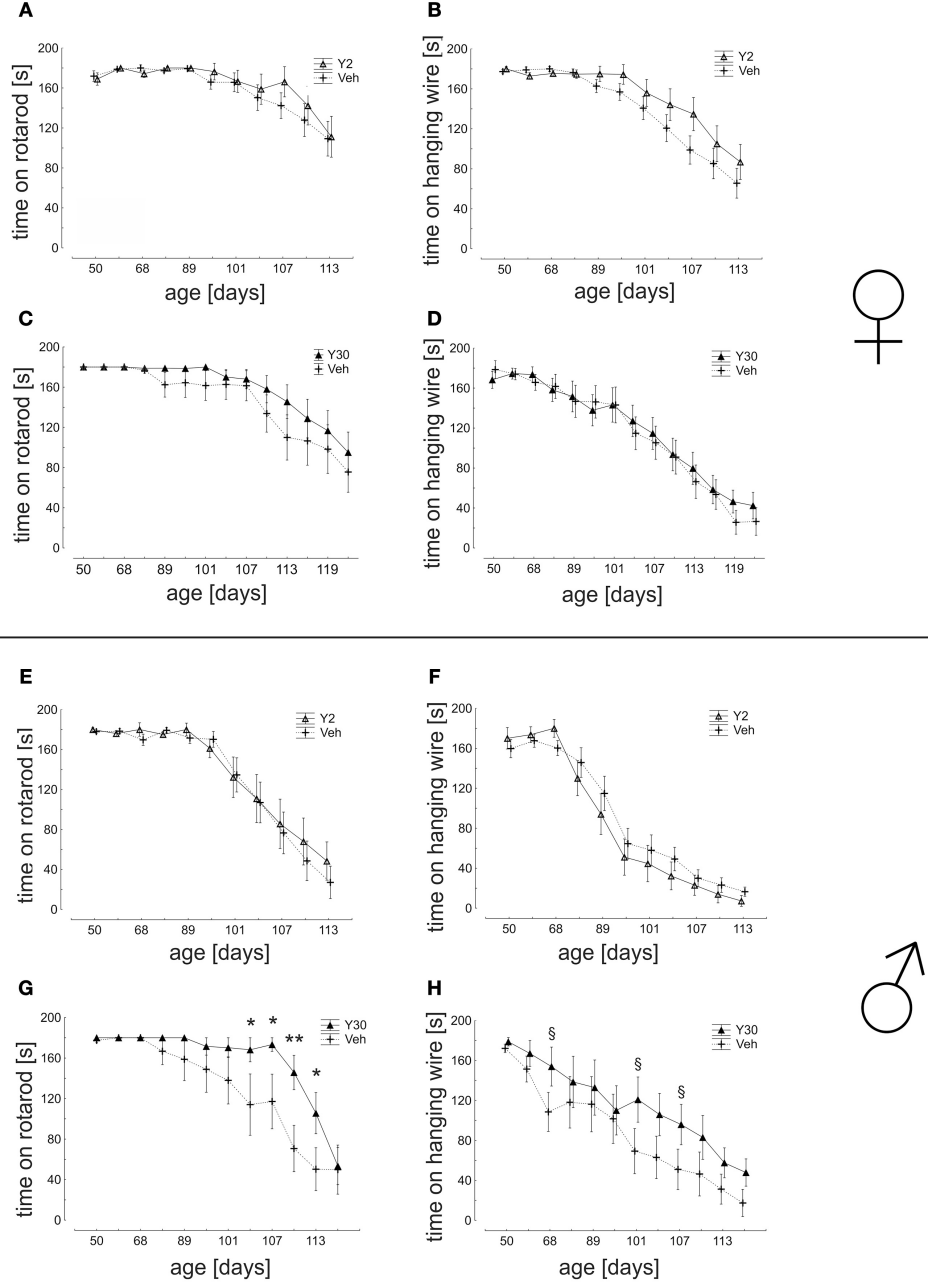
**Motor behavior in SOD1^G93A^ mice treated with 2 or 30 mg/kg body weight Y-27632. (A–D)** Mean time of endurance on the rotarod and in the hanging wire test for female mice treated with Y-27632 from d50 in a dosage of 2 mg/kg **(A,B)** (Y2 *n* = 13; Veh *n* = 18) or 30 mg/kg (C,D) (Y30 *n* = 13; Veh *n* = 12). **(E–H)** Scores for male mice treated with Y-27632 from d50 in a dosage of 2 mg/kg **(E,F)** (Y2 *n* = 10; Veh *n* = 14) or 30 mg/kg **(G,H)** (Y30 *n* = 8; Veh *n* = 8). Statistics was done applying multifactorial ANOVA and *post-hoc* Fisher's test; ^*^P < 0.05; ^**^P < 0.01; in H: § d68 *p* = 0.12; § d101 *p* = 0.08; § d107 *p* = 0.12.

In the analysis of motor coordination and muscle strength as assessed by the ability to remain on a rotating rod, a significant advantage was again detected for male mice treated with Y30 in the time period from d80 until d113 (d104: Veh 114,04 ± 30,24 s; Y30 168,17 ± 11,83 s; d107: Veh 117,13 ± 26,94 s; Y30 173,31 ± 6,69 s; d110: Veh 70,67 ± 22,90 s; Y30 145,79 ± 16,97 s; d113 Veh 50,29 ± 21,19 s; Y30 105,56 ± 20,31 s). Similarly, in the analysis of muscle strength measured by hanging wire the vehicle group performed worse in comparison to the Y30 male group, however, without reaching significance. Animals treated with Y2 did not exhibit improved motor behavior nor did any of the female cohorts (Figure 6).

## Discussion

Motoneuron death and axonal degeneration are critical pathogenic features in the development of human ALS (Fischer et al., 2004). ROCK inhibition by fasudil was previously shown to exert neuroprotective, but also pro-regenerative and axon-stabilizing effects, but fasudil is a rather unspecific drug in comparison to the more specific ROCK inhibitor Y-27632. Therefore, we have now evaluated the therapeutic potential of orally applied Y-27632 in the SOD1^G93A^ mouse model of ALS.

### Pharmacological inhibition of ROCK protects motoneurons in culture

ROCK inhibitors have been applied successfully in a multitude of cell death paradigms. They have shown to attenuate apoptotic cell death of retinal ganglion cells (Lingor et al., 2008; Tura et al., 2009) and prevent dopaminergic cell loss in toxin-based models of Parkinson's disease (Tonges et al., 2012). Recently, Y-27632 was also shown to protect hippocampal neurons against neuronal cell death induced by glutamate *in vitro* and by kainic acid *in vivo* (Jeon et al., 2013).

In a model of kainate-induced glutamate excitotoxicity, our group was able to demonstrate that the ROCK inhibitor fasudil significantly increased the number of surviving MN (Tonges et al., 2014). This neuroprotective effect was now also observed for Y-27632, which reduced the loss of motoneurons in dissociated cell culture. Furthermore, neurite outgrowth was promoted on a permissive substrate—an effect, which is well known for ROCK inhibitors if applied to neuronal cells (Lingor et al., 2008; Tonges et al., 2012).

However, it has to be taken into account that there seems to be an optimal dosage window for Y-27632 *in vitro*—as is known for other ROCK inhibitors, too—because final concentrations of 2 or 50 μM, being fivefold lower or higher than the optimal 10 μM, were not able to elicit a significant outgrowth promoting effect of MN anymore. This could be due to the inhibition of protein kinases other than ROCK which are known for both Y-27632 and fasudil—however, to a much lower extent for Y-27632. In fact, a concentration of 20 μM fasudil is needed to reduce ROCK-II activity *in vitro* to 7% of control and a concentration of 10 μM Y-27632 is needed to reduce ROCK-II activity *in vitro* to 13%. The concentrations of both compounds required for 50% inhibition (IC_50_) of ROCK2 are 1900 nM for fasudil and 800 nM for Y-27632. Most importantly, the activities of several other kinases are much stronger reduced with fasudil (20 μM) (MAPKAP-K1b 37%, MSK1: 19%, PKA: 35%, S6K1 32%) than with Y-27632 (10 μM) (MAPKAP-K1b: 72%, MSK1: 57%; PKA: 91%, S6K1 94%), which may elicit neurobiological side effects albeit being difficult to predict.

### The disease course in SOD1^G93A^ mice is only transiently improved by Y-27632 treatment

In the evaluation of clinical symptoms of disease, we found only a transient improvement for male mice with the high-dose Y30 treatment, which correlated with the transient improvement in motor performance. In contrast, survival of SOD1^G93A^ mice was not significantly prolonged in this study in any group.

In a previous study of our group, we had orally applied the more unspecific ROCK inhibitor fasudil to SOD1^G93A^ mice starting at postnatal day 50 (d50) (Tonges et al., 2014). Here, survival was significantly prolonged with fasudil 30 mg/kg and by trend with fasudil 100 mg/kg. The smaller effect by fasudil 100 mg/kg could be due to a U-shaped dose-response relationship, which we have observed before *in vitro* (Lingor et al., 2007; Tonges et al., 2012). Similar effects of fasudil on survival in the SOD1^G93A^ model have been reported by another group who found a prosurvival response, which was attributed to an attenuation of ROCK activity and an increased level of phospho-Akt (Takata et al., 2013).

Other trials with Y-27632 in models of neurodegenerative disease had shown more favorable results. In a model of Huntington's disease, the ROCK inhibitor Y-27632 improved rotarod performance without influencing survival time at a dosage of 100 mg/kg (Li et al., 2009). The two ROCK inhibitors Y-27632 (30 mg/kg) and fasudil (30 mg/kg) were also beneficially applied in mouse models of SMA, in which they prolonged survival and preserved the integrity of the neuromuscular junction (NMJ). Interestingly, the effect on survival was more pronounced with fasudil than with Y-27632 (Bowerman et al., 2010, 2012).

Overall, the application of Y-27632 was less successful in the SOD1^G93A^ mouse model than that of fasudil. Since 30 mg/kg body weight showed only significant effects on motor function, we cannot exclude that an even higher dosage would be more beneficial. It could also be that the differential inhibitory profile toward other kinases is responsible for the lack of efficacy (Davies et al., 2000). Thus, some of these “off-target” effects on other kinases by fasudil may have been even beneficial to a certain extent in this ALS model.

### Gender-differences of ROCK inhibition with Y-27632 in the SOD1^G93A^ model

It is known that female mice have longer survival times in the SOD1^G93A^ mouse model than males. This fact has also been observed in our study. However, a minor trend to improved survival with Y-27632 is only seen in females treated with 30 mg/kg Y-27632 but not in male mice. Interestingly, the transient improvement of motor function was detected only in male mice on a significant level and was observed only by trend in female mice. This could be due to a more aggressive disease course associated with increased ROCK activity in intermediate stages in male mice, which then benefit more from the Y-27632 application. Supportive of this hypothesis is a study that has been performed in the MPTP model of Parkinson's disease in female mice. If these mice were ovariectomized, an increased loss of dopaminergic neurons after MPTP toxicity was observed, which was shown to be associated with an increase of ROCK activity. Thus, higher ROCK activity correlated to increased vulnerability of dopaminergic neurons to MPTP. When Y-27632 was applied in this model, the increased loss of dopaminergic cells was reversed being as effective as an estrogen-replacement therapy (Rodriguez-Perez et al., 2013).

### Conclusion

The role of ROCK and the therapeutic potential of its inhibition are increasingly studied in models of neurodegenerative disease. However, findings obtained with one ROCK inhibitor may not be generalized to the entire class of ROCK inhibitors, which show high chemical heterogeneity. Whereas the application of fasudil was successfully promoting survival of SOD1^G93A^ mice in two independent studies, this was not found for Y-27632 in two different dosages in the present paradigm. Suboptimal targeting of the therapeutic window as well as the differential inhibition of other protein kinases may contribute to these differences. As most studies have been performed in female mice, a gender-dependent responsiveness to ROCK inhibition may play an additional role. Our study thus supports the need for thorough evaluation of ROCK inhibitors in models of neurodegenerative disease in order to better characterize their effects and thereby optimally prepare their translation into human clinical trials.

### Conflict of interest statement

The authors declare that the research was conducted in the absence of any commercial or financial relationships that could be construed as a potential conflict of interest.

## Abbreviations

ANOVA: analysis of variance
MAPKAP-K1b: MAPK-activated protein kinase-1b
MN: motoneuron
MSK1: mitogen- and stress-activated protein kinase 1
PKA: cAMP-dependent protein kinase
ROCK: rho kinase
S6K1: p70 ribosomal protein S6 kinase
SMA: spinal muscular atrophy
SOD1^G93A^: superoxide dismutase 1 G93A transgenic
Veh: vehicle
Y2: Y-27632 2 mg/kg body weight
Y30: Y-27632 30 mg/kg body weight.
