# Supplementary material for: The rho kinase inhibitor Y-27632 improves motor performance in male SOD1G93A mice
Source: Front Neurosci. 2014 Oct 7;8:304. doi: 10.3389/fnins.2014.00304 (PMC4187656; doi:10.3389/fnins.2014.00304)
Supplement: Supplementary file 1 [file DataSheet1.DOCX]

***Günther el al.:***

***The rho kinase inhibitor Y-27632 improves motor performance in male SOD1G93A mice***

**Supplementary Data**


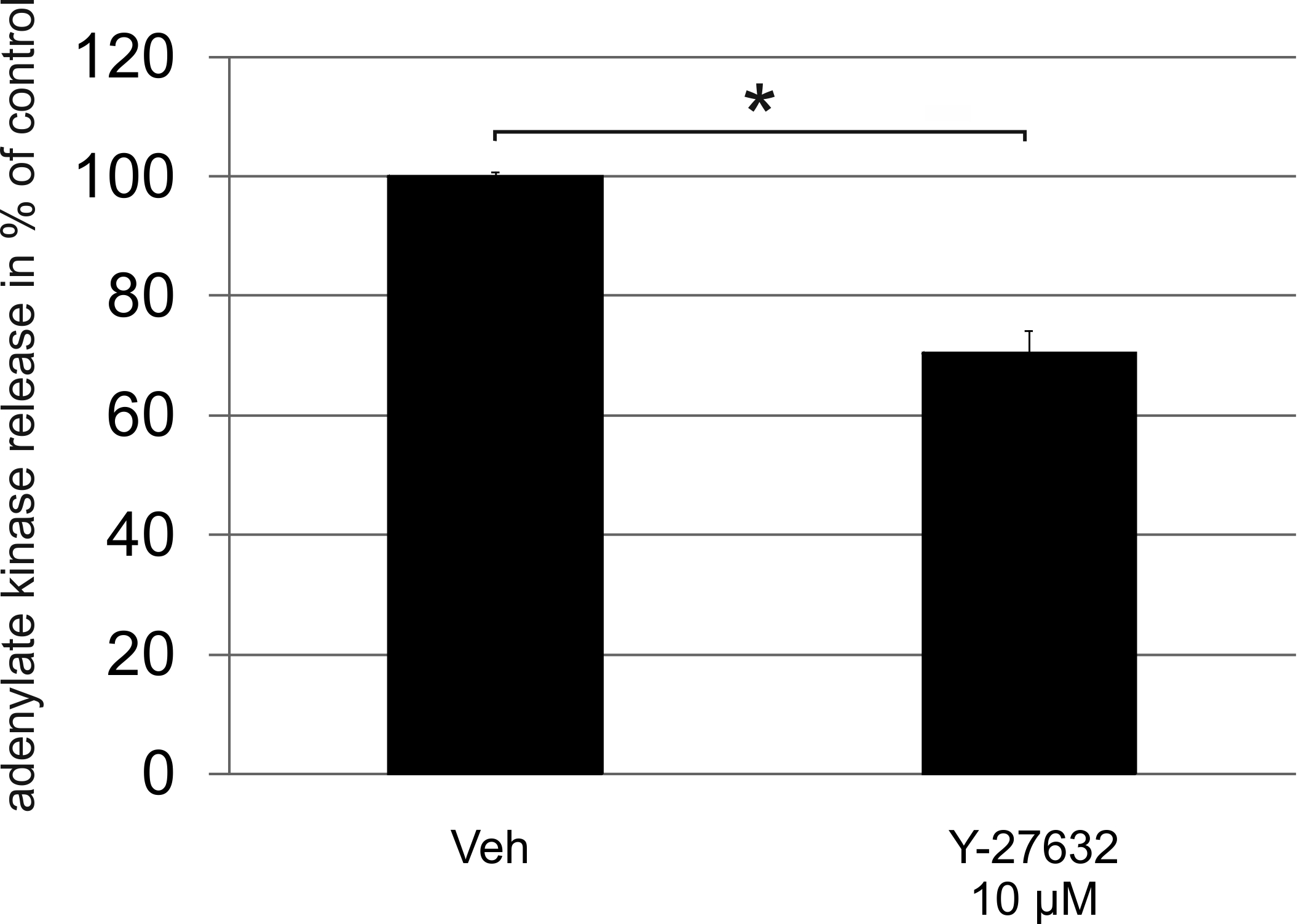


**Suppl. Fig. 1:** Cell toxicity (ToxiLight^TM^) assays of motoneuron cultures at DIV4. The histogram shows relative cell toxicity as measured by the release of adenylate kinase in cultures treated with vehicle (Veh) or with Y-27632 (10 µM). Bars represent means ± SEM. *p<0.05 according to unpaired Student’s t-test.


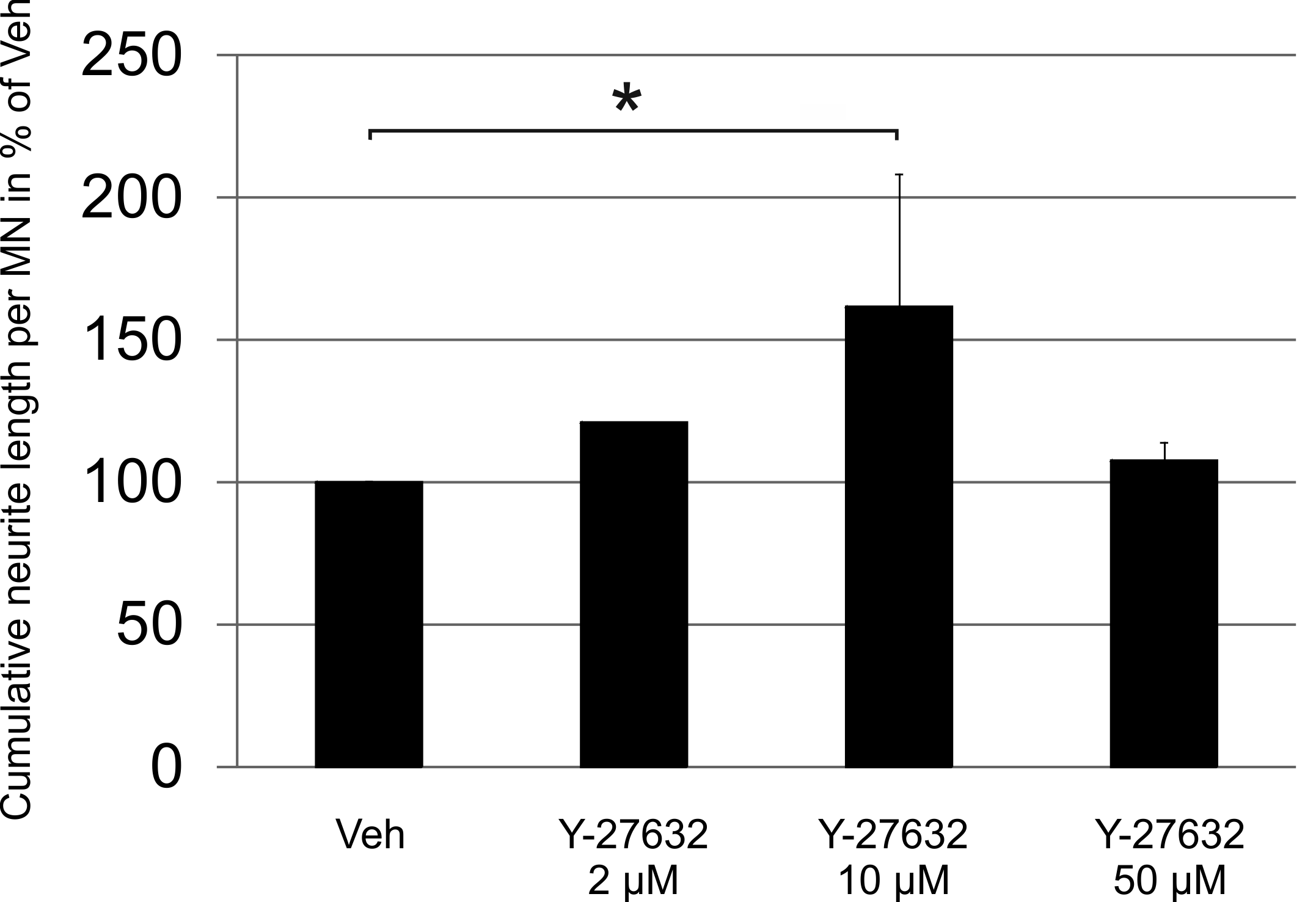


**Suppl. Fig. 2:** Relative cumulative neurite outgrowth of motoneurons (MN) in vitro treated with different doses of Y-27632 for four days in culture (n = 2 MN cultures, bars represent means ± SEM; *P < 0.05, according ANOVA and Dunnett´s test).


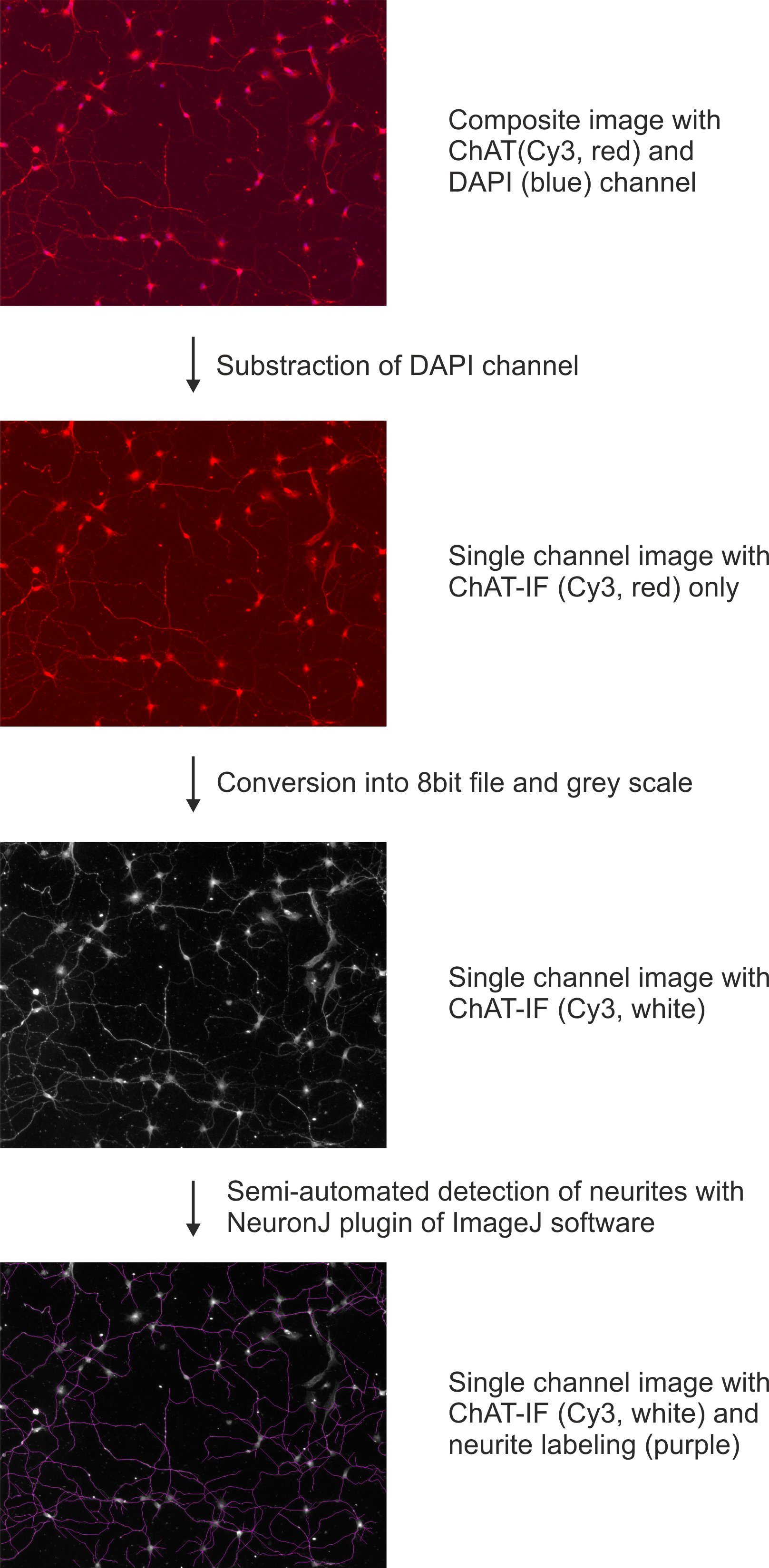


**Suppl. Fig. 3:** Workflow for the measurement of neurite length in ChAT-immunopositive motoneurons in vitro.


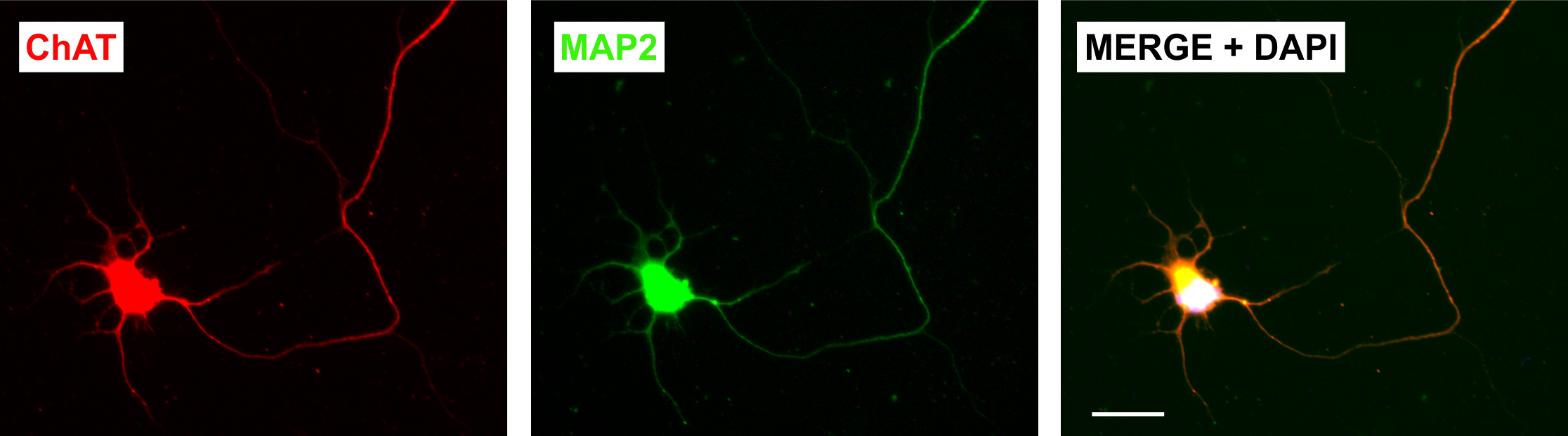


**Suppl. Fig. 4**: Immunolabeling of a motoneuron with an antibody directed against ChAT (red) and against MAP2 (green). The ChAT staining labels the entire neurite as does MAP2. Scale bar 20 µm.
